# 10-year follow-up study on medical expenses and medical care use according to biological age: National Health Insurance Service Health Screening Cohort (NHIS-HealS 2002~2019)

**DOI:** 10.1371/journal.pone.0282466

**Published:** 2023-03-02

**Authors:** Chul-young Bae, Bo-seon Kim, Kyung-hee Cho, In-hee Kim, Jeong-hoon Kim, Ji-hyun Kim

**Affiliations:** 1 MediAge Research Center, Seongnam-si, Gyeonggi-do, Republic of South Korea; 2 Department of Family Medicine, National Health Insurance Service Ilsan Hospital, Goyang-si, Gyeonggi-do, Republic of South Korea; KMCH Institute of Health Sciences and Research, INDIA

## Abstract

**Objectives:**

The world is witnessing a sharp increase in its elderly population, accelerated by longer life expectancy and lower birth rates, which in turn imposes enormous medical burden on society. Although numerous studies have predicted medical expenses based on region, gender, and chronological age (CA), any attempt has rarely been made to utilize biological age (BA)—an indicator of health and aging—to ascertain and predict factors related to medical expenses and medical care use. Thus, this study employs BA to predict factors that affect medical expenses and medical care use.

**Materials and methods:**

Referring to the health screening cohort database of the National Health Insurance Service (NHIS), this study targeted 276,723 adults who underwent health check-ups in 2009−2010 and kept track of the data on their medical expenses and medical care use up to 2019. The average follow-up period is 9.12 years. Twelve clinical indicators were used to measure BA, while the total annual medical expenses, total annual number of outpatient days, total annual number of days in hospital, and average annual increases in medical expenses were used as the variables for medical expenses and medical care use. For statistical analysis, this study employed Pearson correlation analysis and multiple regression analysis.

**Results:**

Regression analysis of the differences between corrected biological age (cBA) and CA exhibited statistically significant increases (*p*<0.05) in all the variables of the total annual medical expenses, total annual number of outpatient days, total annual number of days in hospital, and average annual increases in medical expenses.

**Conclusions:**

This study quantified decreases in the variables for medical expenses and medical care use based on improved BA, thereby motivating people to become more health-conscious. In particular, this study is significant in that it is the first of its kind to predict medical expenses and medical care use through BA.

## Introduction

The growth in the total population of the elderly (defined as people aged 65 and above), accelerated by increasing life expectancy and declining fertility, is a global phenomenon. Based on the definitions proposed by international organizations, such as the UN and the OECD, a society whose elderly population accounts for more than 7% of its total population is referred to as an *aging society*, while those whose elderly population represent more than 14% and 20% are referred to as an *aged society* and a *super-aged society*, respectively. The US became an aging society in 1942, and Japan in 1970. Korea joined them in 2000 [1]. By the end of September 2021, the registered resident population aged 65 and above in Korea stood at 8.53 million, constituting more than 16.4% of the nation’s total population (51.82 million). Given the continued increase in its elderly population, Korea is expected to become a super-aged society in 2025, when its elderly would have accounted for approximately 20.3% of the total population [2].

Korea’s rapidly aging population has imposed an enormous medical burden on society. According to major health insurance statistics in 2020, the total medical expenses nationwide amounted to KRW86.1 trillion, a 10.5% increase over the previous year [3]. What is worth noting is that the annual medical expenses of senior citizens (per capita) are reported to be growing faster than those of other age groups [4]. Moreover, medical expenses in Korea grows at a rate higher than that in other countries; chances are Korea’s population aging will increase the nation’s medical expenses at an accelerated rate [5–10].

A review of the existing literature on medical expenses reveals that previous studies do not go deeper than simply confirming the different rates of increase in medical expenses over time across various age groups [11–16]. Germany and Norway reported high growth rates in medical expenses among their elderly people and used the term “age steepening” [17,18]. Notably, there are limited longitudinal studies on medical expenses for the elderly over 65 years of age. A research paper that consulted the Korea Health Panel to examine medical expenses for Korean senior citizens aged 65 and above for the period 2008−2013 verified the linear growth of such medical expenses for the elderly over time [19]. A research project conducted in China in 2017 highlighted the effect of increasing CA on medical expenses, advising the nation’s elderly people to take care of themselves to reduce their medical expenses and conserve national medical resources [20].

Most of these previous studies, which focus on the factors attributable to increases in medical expenses, utilized survey data to predict medical expenses and the frequency of outpatient and emergency room visits by region, gender, and CA [19,21–23]. Some studies centered on the elderly population added economic activity and the number of chronic diseases to the list of aforementioned factors affecting medical expenses [24–27].

CA is a widely used indicator of aging. However, the life expectancy of people with the same or similar CA can still differ significantly, depending on heredity, lifestyle, and living environment. An individual aged 50 may have a physical function of those aged 60, and many people look older or younger compared to others at the same CA. Thus, it is well known that CA is not an optimal indicator for the aging progress [28,29].

To supplement the disadvantages of CA as an indicator, BA is frequently used to predict death and aging-related diseases. As aging is closely related to death and onset of disease, BA-based predictions yield more statistically significant results [30–35]. While extensive research has been conducted to predict death and disease outbreaks through BA, little research has been done to estimate the factors affecting medical expenses and medical care use through BA [36,37].

The present study examined the 10-year trend (2009−2019) of data on annual medical expenses and annual medical care use by gender and age group, and employed BA to estimate the total annual medical expenses, total annual number of outpatient days, and total annual number of days in hospital for the baseline (2009−2010). Also using BA-based total annual medical expenses for a 10-year period (2009−2019), this study predicted increases in annual medical expenses.

## Materials and methods

### Research subjects

This study used the health screening cohort database of the NHIS, which was followed up from 2002 to 2019, with 514,867 individuals, or 10% of the 5.15 million citizens who remained eligible for national health insurance as of December 2002. This data is a secondary source that has been deidentified. Therefore, since consent cannot be obtained and identification such as personal information is not included, it is not subject to written consent.

**Fig 1:**
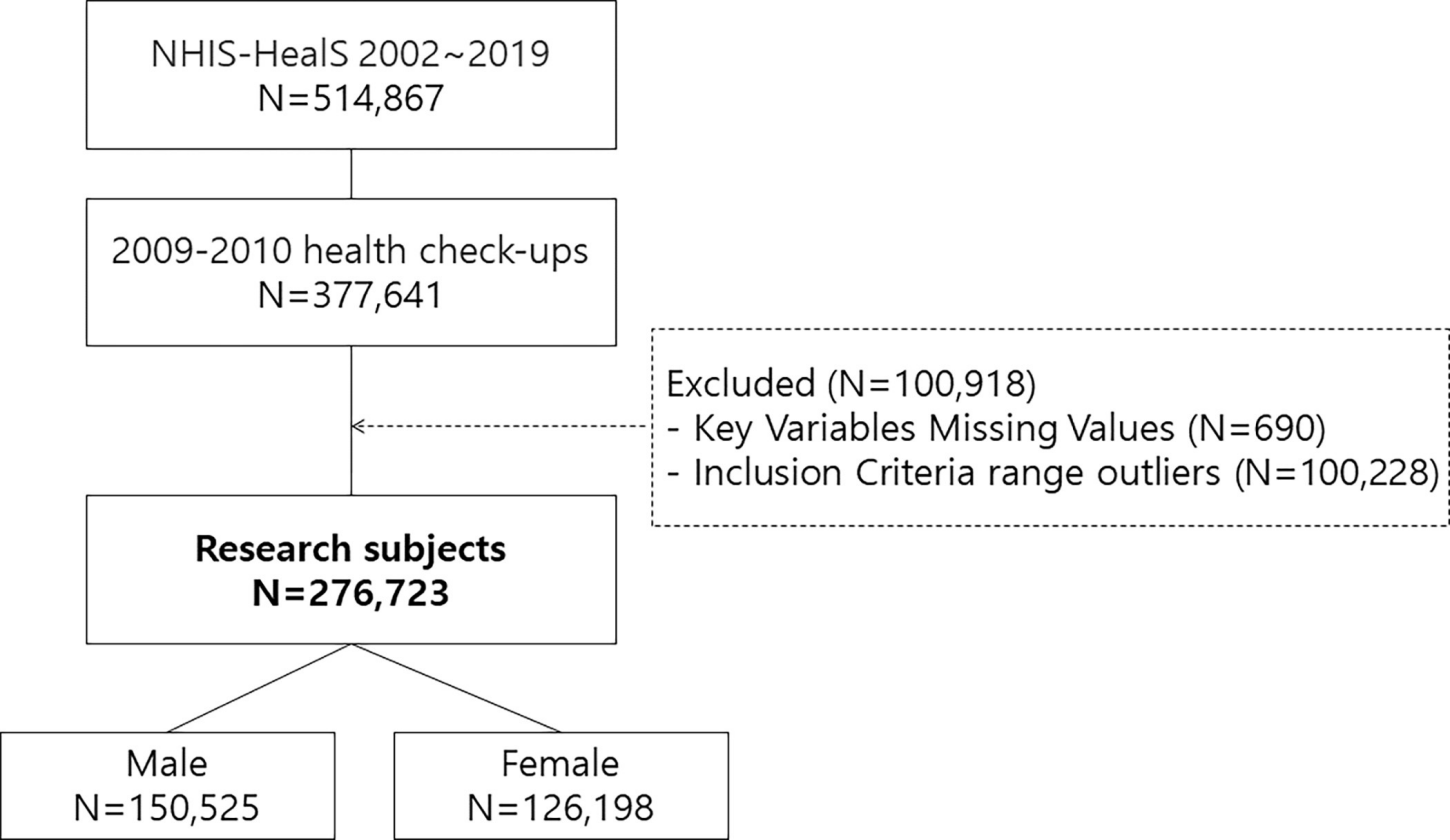
Flowchart detailing the subject selection process.

Waist circumference, one of the major variables for BA, was officially introduced in 2009 as part of the biennial national health check-ups, so we selected 377,641 subjects (who underwent national health check-ups), with 2009−2010 as the baseline. After excluding 690 subjects with missing values for key variables, we applied the inclusion criteria, as shown in Table 1 and adopted by the BA-related study [34], to excluded another 100,228. A total of 276,723 subjects consisting of 150,525 males and 126,198 females were finally selected (Fig 1). We then kept track of the data on the subjects’ medical expenses and medical care use from the baseline to 2019. The average follow-up period is 9.12 years (9.27 years for men and 8.88 years for women). The longest follow-up period is 9.92 years (9.92 years for men and 9.89 years for women).

**Table 1:** Inclusion criteria for the study parameters.

Parameters	Inclusion criteria	
	Lower Limit(≤)	Upper Limit(<)
**HT**^**1**^ **(cm)**	NA	
**WC**^**2**^ **(cm)**	60.00	105.00
**SBP**^**3**^ **(mmHg)**	80.00	160.00
**FBS**^**4**^ **(mg/dL)**	50.00	140.00
**TC**^**5**^ **(mg/dL)**	50.00	260.00
**TG**^**6**^ **(mg/dL)**	50.00	400.00
**HDL-C**^**7**^ **(mg/dL)**	20.00	90.00
**HMG**^**8**^ **(mg/dL)**	10.00	18.00
**Cr**^**9**^ **(mg/dL)**	0.40	2.00
**AST**^**10**^ **(IU/L)**	-	60.00
**r-GTP**^**11**^ **(IU/L)**	-	150.00

^1^HT: Height

^2^WC: Waist circumference

^3^SBP: Systolic blood pressure

^4^FBS: Fasting blood sugar

^5^TC: Total cholesterol

^6^TG: Triglycerides

^7^HDL-C: High-density lipoprotein

^8^HMG: Hemoglobin

^9^Cr: Creatinine

^10^AST: Aspartate aminotransferase

^11^r-GTP: Gamma-glutamyl transpeptidase.

This study was approved exempt from review by the Institutional Review Board of the National Health Insurance Service Ilsan Hospital (NHIMC 2021-01-003). The reason for the exemption from the Institutional Review Board is that the data that could not confirm the subject’s identity was analyzed in this study.

### Measurement of BA

This study selected 12 variables out of 15 examination items used by the NHIS as clinical indicators to measure BA. These variables correspond to the variables used by [34] to build a BA model. Anthropometric markers include HT, WC, and SBP, while blood biomarkers encompass FBS, TC, TG, HDL-C, HMG, Cr, AST, r-GTP, and e-GFR. Furthermore, this study utilized the model created by [34] for cBA, the indicators used to measure BA. The difference between cBA and CA function as an independent variable against the baseline model is given in Eq (1):

CorrectedBAinMen=−127.6−0.19×(HT)+0.88×(WC)+0.46×(SBP)+0.40×(FBS)+0.57×(HMG)−0.17×(eGFR)+0.62×(AST)+0.65×(Age)

CorrectedinBAinWomen=−0.01−0.45×(HT)+0.49×(WC)+0.24×(SBP)+0.22×(FBS)+0.07×(TC)+0.07×(TG)−0.18×(HDL−C)−0.07×(eGFR)+0.44×(AST)+0.20×(γ−GTP)+0.36×(Age)

### Medical expenses and medical care use

To analyze the 10-year trend (2009−2019) of the data on annual medical expenses and medical care use by gender and age group, this study utilized the total annual medical expenses, total annual number of outpatient days, and total annual number of days in hospital. For the model that estimates the total annual medical expenses, annual number of outpatient days, and annual number of days in hospital for the baseline (2009−2010) depending on the differences between cBA and CA, this study treated the annual data on medical expenses, number of outpatient days, and number of days in hospital as dependent variables. More specifically, we borrowed related data from the NHIS treatment database: the total approved medical care expenses to calculate medical expenses, the number of days of care to calculate the number of outpatient days, and the number of hospital visits and days in hospital to calculate the number of days in hospital.

### Statistical analysis

The general characteristics of the subjects are represented by the average and standard deviation. Multiple regression analysis was applied on the differences between cBA and CA to predict annual medical expenses and annual medical care use, as well as increases in annual medical expenses. This study used R Studio version 3.3.3 to perform the above analyses with the significant level set at p<0.05.

## Results

### General characteristics of the study subjects

For this research, 276,723 subjects (150,525 men and 126,198 women) were selected. The average age of all the subjects was 58.91 ± 8.8, and the general characteristics of their clinical indicators are shown in Table 2.

**Table 2:** General characteristics of the subjects.

Parameters	Mean±SD^1^		
	All (N = 276,723)	Male (N = 150,525)	Female (N = 126,198)
**AGE (years)**	58.91 ± 8.8	58.36 ± 8.7	59.57 ± 8.9
**HT**^**2**^ **(cm)**	160.98 ± 8.9	167.22 ± 5.9	153.82 ± 5.8
**WC**^**3**^ **(cm)**	81.44 ± 7.7	83.98 ± 6.9	78.42 ± 7.4
**SBP**^**4**^ **(mmHg)**	123.36 ± 13.2	124.28 ± 12.6	122.26 ± 13.7
**FBS**^**5**^ **(mg/dL)**	96.22 ± 13.3	97.54 ± 13.8	94.65 ± 12.6
**TC**^**6**^ **(mg/dL)**	195.08 ± 30.9	191.51 ± 30.9	199.34 ± 30.3
**TG**^**7**^ **(mg/dL)**	130.92 ± 64.5	137.01 ± 67.7	123.66 ± 59.8
**HDL-C**^**8**^ **(mg/dL)**	52.89 ± 12.0	51.03 ± 11.7	55.11 ± 12.1
**HMG**^**9**^ **(mg/dL)**	13.83 ± 1.4	14.63 ± 1.2	12.87 ± 1.0
**Cr**^**10**^ **(mg/dL)**	0.91 ± 0.2	1.01 ± 0.2	0.79 ± 0.2
**AST**^**11**^ **(IU/L)**	22.75 ± 7.2	25.3 ± 7.4	23.65 ± 6.8
**r-GTP**^**12**^ **(IU/L)**	30.38 ± 22.4	37.84 ± 25.0	21.48 ± 14.5
**eGFR**^**13**^ **(mL/min/1.73 m2)**	83.79 ± 18.8	84.44 ± 18.7	83.03 ± 18.1

^1^SD, standard deviation

^2^HT: Height

^3^WC: Waist circumference

^4^SBP: Systolic blood pressure

^5^FBS: Fasting blood sugar

^6^TC: Total cholesterol

^7^TG: Triglycerides

^8^HDL-C: High-density lipoprotein

^9^HMG: Hemoglobin

^10^Cr: Creatinine

^11^AST: Aspartate aminotransferase

^12^r-GTP: Gamma glutamyl transpeptidase

^13^eGFR: Estimated glomerular filtration rate.

### Baseline (2009−2010) medical expenses and medical care use by gender and age group

To compare medical expenses and medical care use by gender and age group, this study used the baseline (2009–2010) average of total annual medical expenses, total annual number of outpatient days, and total annual number of days in hospital. The averages of all three categories turned out to be higher among women than men. For both men and women, average of total annual medical expenses, total annual number of outpatient days, and total annual number of days in hospital grew at as the age group on the rised (Table 3).

**Table 3:** Medical expenses and medical care use by gender and age group.

Parameters	Age group	Mean±SD^1^	
		Male (N = 150,525)	Female (N = 126,198)
**Medical expenses**	**All Ages**	732,839 ± 1,935130	848,816 ± 1,703,491
	**40≤Age<50**	402,939 ± 1,020,294	507,069 ± 1,168,831
	**50≤Age<60**	541,020 ± 1,504,036	648,382 ± 1,364,656
	**60≤Age<70**	865,734 ± 2,133,483	975,371 ± 1,836,367
	**70≤Age**	1,402,734 ± 2,961,328	1,348,348 ± 2,281,137
**Number of outpatient days**	**All Ages**	35.8 ± 81.6	43.3 ± 73.0
	**40≤Age<50**	18.7 ± 44.1	21.5 ± 37.7
	**50≤Age<60**	24.1 ± 54.1	29.3 ± 47.6
	**60≤Age<70**	43.2 ± 98.3	48.6 ± 73.2
	**70≤Age**	75.0 ± 122.1	83.1 ± 113.7
**Number of days in hospital**	**All Ages**	20.8 ± 25.2	28.0 ± 27.7
	**40≤Age<50**	12.2 ± 14.9	16.7 ± 16.9
	**50≤Age<60**	15.6 ± 17.5	21.7 ± 21.0
	**60≤Age<70**	24.7 ± 26.4	31.9 ± 28.5
	**70≤Age**	38.6 ± 37.8	43.9 ± 36.7

^1^SD, standard deviation.

### Ten-year trend (2009–2019) of medical expenses and medical care use by gender and age group

**Fig 2:**
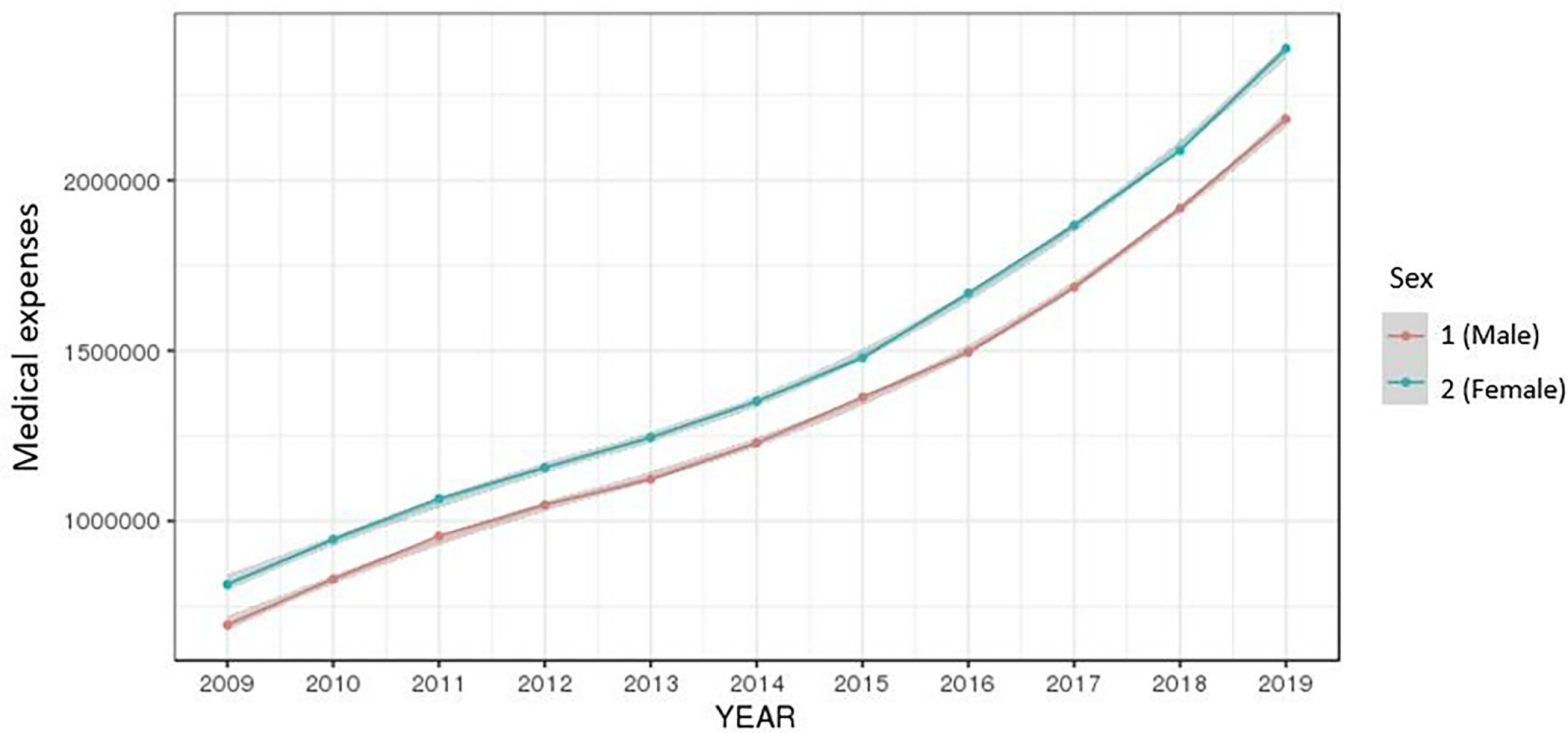
Ten-year trend of medical expenses by gender.

**Fig 3:**
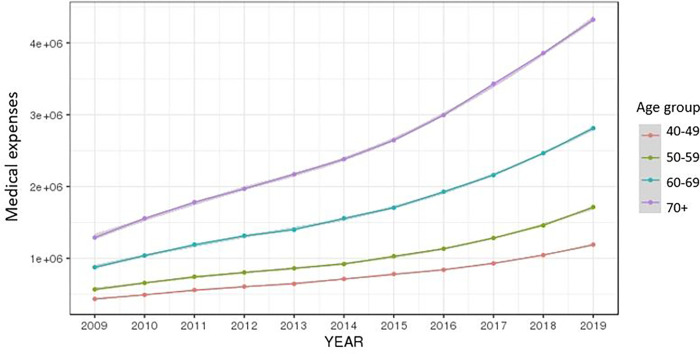
Ten-year trend of medical expenses by age group.

The total annual medical expenses, total annual number of outpatient days, and total annual number of days in hospital for the period 2009–2019 were averaged by gender and age group, and were then rendered into a graph. The average of the total medical expenses by year was on a steady rise for both men and women and increased at higher rates in older age groups (Figs 2 and 3).

**Fig 4:**
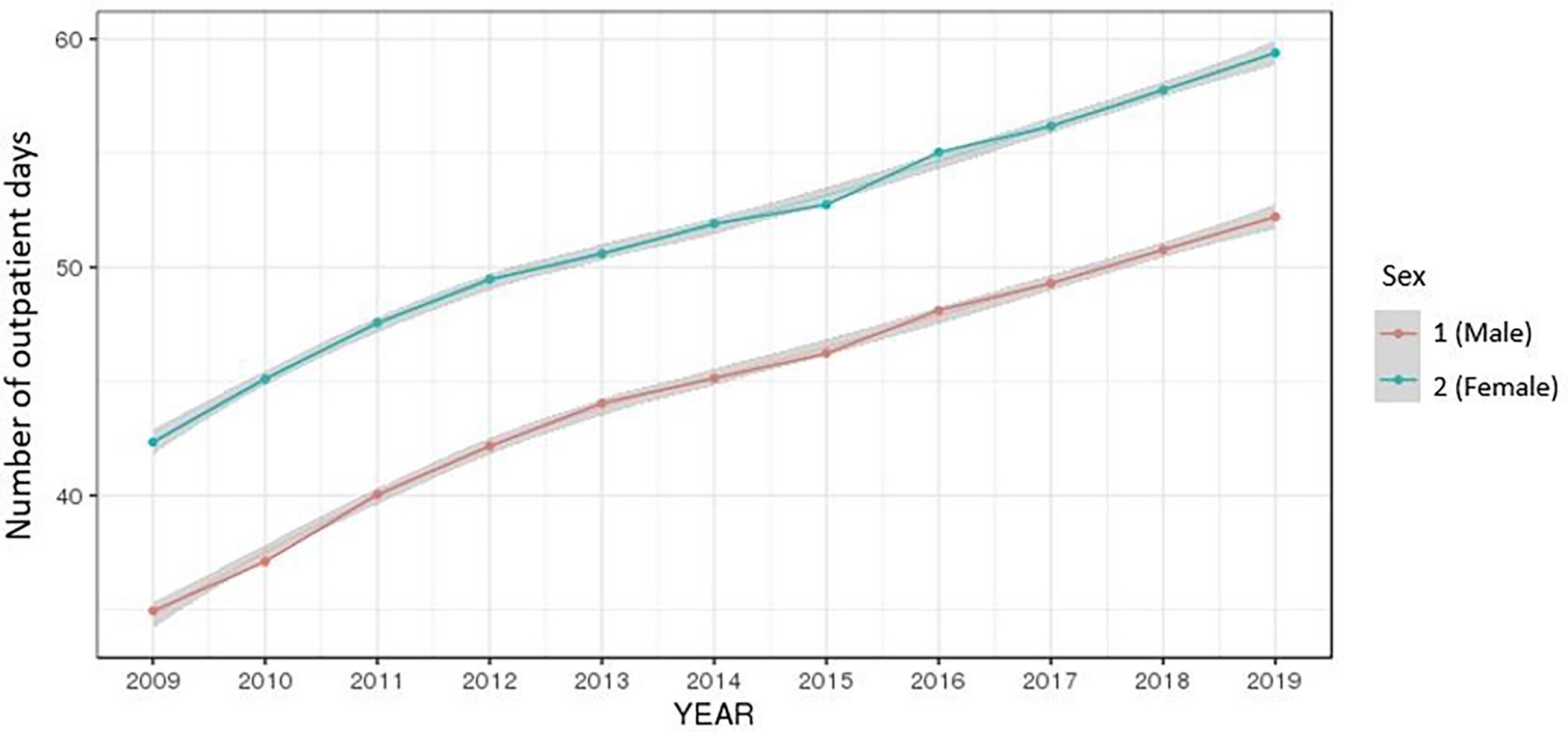
Ten-year trend of the number of outpatient days by gender.

**Fig 5:**
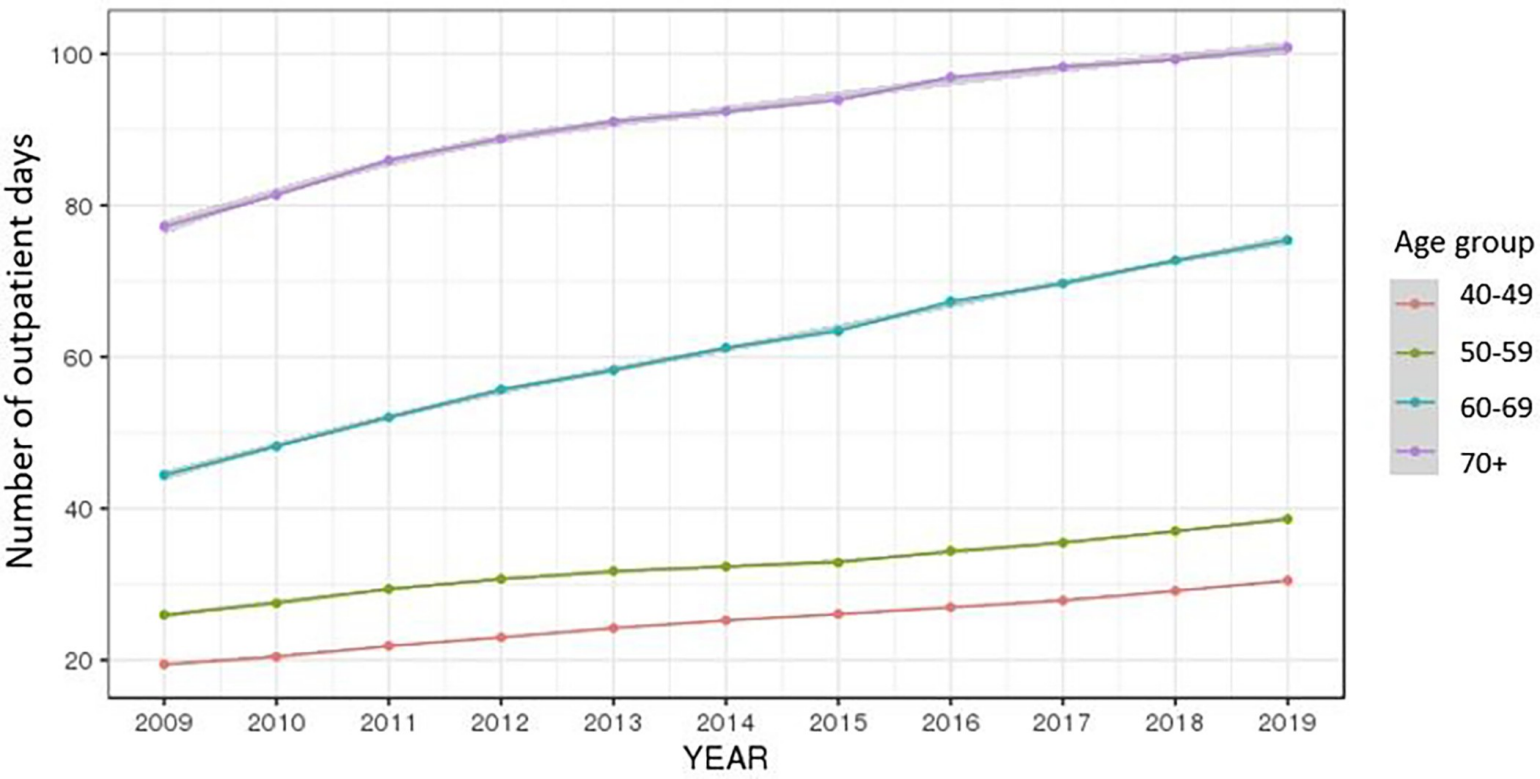
Ten-year trend of the number of outpatient days by age group.

The average of the total number of outpatient days by year continued to rise for both men and women and increased at higher rates in older age groups, diminishing after 70 (Figs 4 and 5).

**Fig 6:**
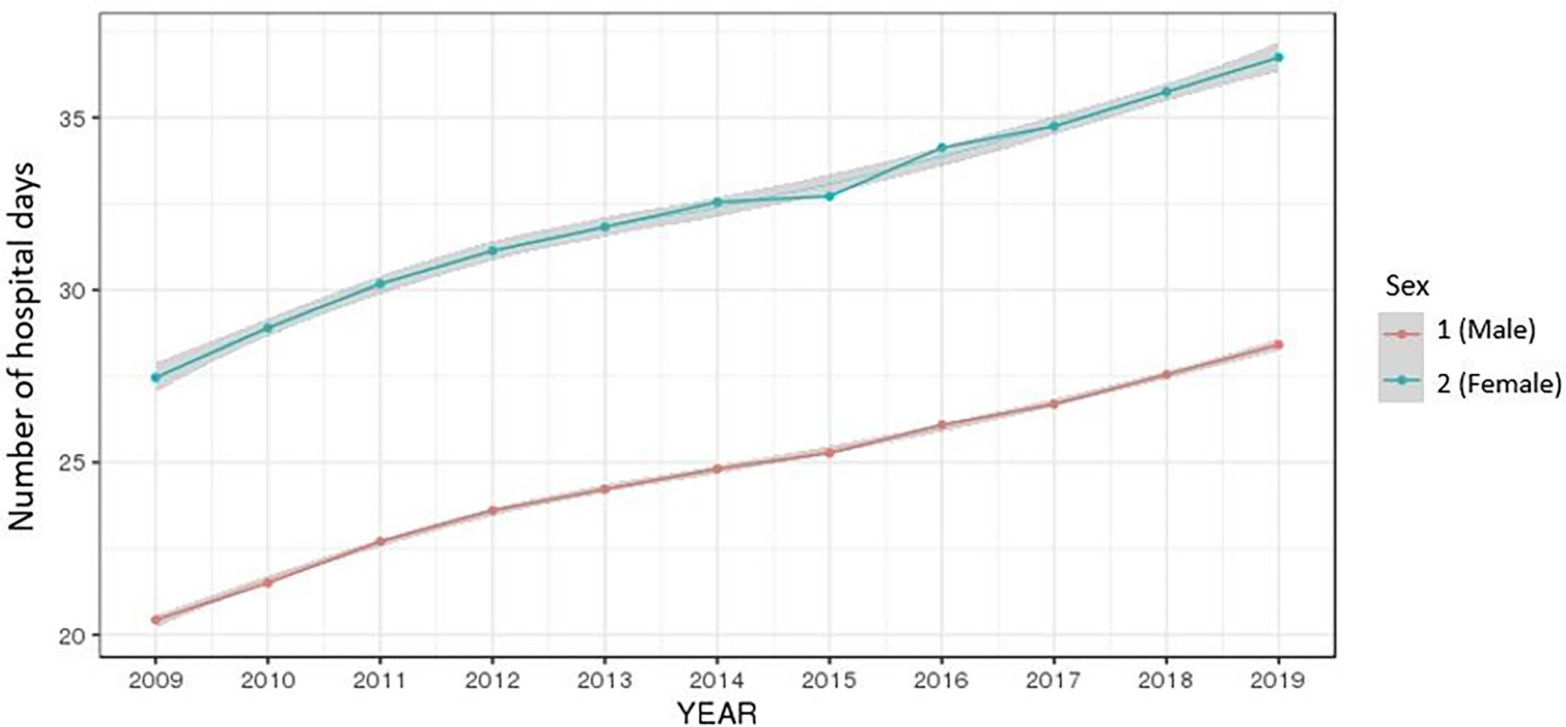
Ten-year trend of the number of days in hospital by gender.

**Fig 7:**
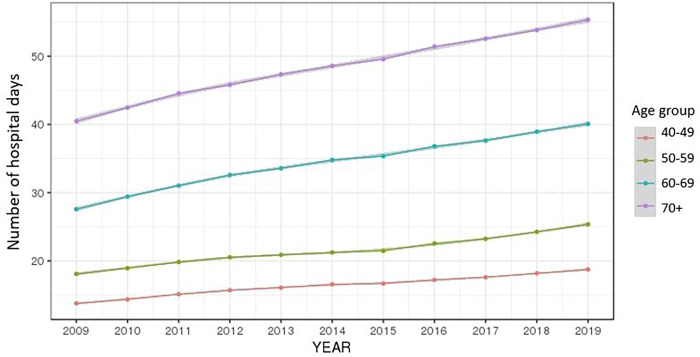
Ten-year trend of the number of days in hospital by age group.

The average of the total number of days in hospital by year continued in an upward trend for both men and women and increased at higher rates the higher the age (Figs 6 and 7).

### Prediction of annual medical expenses and medical care use depending on the differences between cBA and CA

This study estimated the total annual medical expenses, total annual number of outpatient days, and total annual number of days in hospital for the baseline depending on the differences between cBA and CA.

We found that for all the subjects regardless of gender, the higher the difference between cBA and CA, the higher the total annual medical expenses, total annual number of outpatient days, and total annual number of days in hospital are to increase significantly. In particular, per the difference between cBA and CA, the increase in total medical expenses per year, outpatient days per year, and total hospitalization days per year was all higher for women than for men (Table 4).

**Table 4:** Prediction results of annual medical expenses and medical care use depending on the differences between cBA and CA.

Parameters	Sex	Prediction result
**Medical expenses**	**All**	Y = -1,326,669 + 46,072 x (cBA—Age) + 35,694 x (Age)
	**Male**	Y = -1,463,024 + 32,916 x (cBA—Age) + 37,437 x (Age)
	**Female**	Y = -1,140,626 + 64,494 x (cBA—Age) + 33,126 x (Age)
**Number of outpatient days**	**All**	Y = -97.0213 + 2.7635 x (cBA—Age) + 2.3019 x (Age)
	**Male**	Y = -91.6919 + 1.3800 x (cBA—Age) + 2.1742 x (Age)
	**Female**	Y = -100.9725 + 3.7738 x (cBA—Age) + 2.4058 x (Age)
**Number of days in hospital**	**All**	Y = -38.2707 + 1.6036 x (cBA—Age) + 1.0535 x (Age)
	**Male**	Y = -38.2761 + 0.6205 x (cBA—Age) + 1.0080 x (Age)
	**Female**	Y = -35.5955 + 2.0417 x (cBA—Age) + 1.0581 x (Age)

*p<0.05.

### Prediction of average annual increases in medical expenses depending on the differences between cBA and CA

Based on the total medical expenses by year for the period 2009–2019, this study estimated average annual increases in medical expenses depending on the differences between cBA and CA. The average annual increases in medical expenses is given in Eq (2):

$$Average\;annual\;increases\;in\;medical\;expenses=\frac{Total\;medical\;expenses\;in\;the\;last\;year-total\;medical\;expenses\;in\;the\;initial\;year}{the\;last\;year-the\;initial\;year}$$

The average annual increases in medical expenses was higher for women than for men. For both men and women, average annual increases in medical expenses grew at as the age group on the rised (Table 5).

**Table 5:** Average annual increases in medical expenses by gender and age group.

Parameters	Age group	Mean±SD^1^	
		Male (N = 150,525)	Female (N = 126,198)
**Average increases in annual medical expenses**	**All Ages**	165,286 ± 664,129	167,968 ± 604,554
	**40≤Age<50**	76,500 ± 393,543	81,894 ± 331,341
	**50≤Age<60**	124,438 ± 597,490	117,756 ± 483,927
	**60≤Age<70**	216,970 ± 740,820	193,270 ± 630,349
	**70≤Age**	331,672 ± 932,800	328,362 ± 913,387

^1^SD, standard deviation.

As is the case with annual medical expenses and medical care use, for all the subjects regardless of gender, the higher the difference between cBA and CA, the average annual increases in medical expenses grew significantly. For each increase in the difference between cBA and CA, the average annual increases in medical expenses for men was 1.77 times greater than that for women (Table 6).

**Table 6:** Prediction results of average annual increases in medical expenses depending on the differences between cBA and CA.

Parameters	Sex	Prediction results
**Average annual increases in medical expenses**	**All**	Y = -407,975 + 9,055 x (cBA—Age) + 9,805 x (Age)
	**Male**	Y = -422,340 + 12,295 x (cBA—Age) + 10,151 x (Age)
	**Female**	Y = -400,620 + 6,934 x (cBA—Age) + 9,583 x (Age)

*p<0.05.

## Discussion

Utilizing the cohort data of 276,723 individuals over 10 years, this study analyzed the 10-year trend (2009–2019) of annual medical expenses and annual medical care use by gender and age group and estimated the total annual medical expenses, total annual number of outpatient days, and total annual number of days in hospital for the baseline depending on the differences between cBA and CA. This study predicted the growth in annual medical expenses depending on the differences between cBA and CA using total medical expenses by year for the period 2009–2019.

The results of this study are as follows. The higher the difference between cBA and CA, the higher the total annual medical expenses, total annual number of outpatient days, and total annual number of days in hospital are to increase significantly. Notably, per increasing the difference between cBA and CA, women saw their annual medical expenses increase 1.95 times more than those of men. This means that differences between cBA and CA have a greater impact on the increase in women’s annual medical expenses than that in men’s. Per increasing the difference between cBA and CA, increases in the annual number of outpatient days and annual number of days in hospital were 2.73 times and 3.29 times higher for women than for men, respectively. A way to interpret this is that differences between cBA and CA exert greater impact on women than on men in terms of increases in total annual number of outpatient days and total annual number of days in hospital.

Furthermore, depending on the differences between cBA and CA, the growth in annual medical expenses increased significantly. Per increasing the difference between cBA and CA, male subjects saw their annual medical expenses grow by about 1.77 times more than those of their female counterparts. It’s meaning that the differences between cBA and CA influence men more than women in terms of annual medical expense growth.

Previous studies mainly used survey data to explore the factors that affect medical expenses growth. A study that consulted extensive US survey data confirmed the effect of factors, such as region, gender, race, and CA, on medical expenses, as well as on the frequency of emergency room and outpatient visits [21]. In Korea, a study using the 2013 Korea Health Panel Survey predicted medical expenses through subjective social class perception, income level, disability, and CA [22]. An exhaustive study conducted in Hubei, China in 2014 identified determinants, such as CA, disease type, and hospitalization, to explain annual medical expenses [23]. Another research, which delved into the potential predictors for medical expenses of the elderly population for the period 2008–2016, proposed the presence of a spouse, economic activities, and number of chronic diseases as key medical expense-related predictors [31].

The above research mostly focused on the variables of CA and the survey data to predict medical expenses. In contrast, the present study distinguishes itself from these studies in that it is the first of its kind to employ cBA, an indicator that represents health and aging, in estimating the factors of medical expenses and medical care use.

Through this study, even with the same gender and CA, medical expenses and medical care use factors can be predicted in a customized way according to the biological age that reflects the state of health and aging. Moreover, this study employed specific cBA which incorporates the variables of 12 health screening items covered by NHIS, so that the annual medical expenses, annual number of outpatient days, and annual number of days in hospital, as well as increases in annual medical expenses, become predictable whenever national health check-up data is available. Since no additional survey is required, it is possible to conveniently provide customized prediction information to those who have national health check-up. In addition, it is expected to be able to motivate health care by quantitatively presenting the increase in medical expenses and medical care use factors according to the BA.

In the analysis, non-covered items and related medical expenses were not included because the health screening cohort database of NHIS was utilized for this study. Moreover, this study did not take disease types into account, although some studies [24–27] have underscored the causal relationship between disease type and medical expenses. For future studies, analyses of non-covered medical expenses and predictions of detailed medical expenses by disease type will present far more meaningful and significant analytical results.

## Conclusions

This study conducted a 10-year trend analysis of annual medical expenses and annual medical care use by gender and age group based on the cohort data over 10 years. Furthermore, based on the differences between cBA and CA, analyses were performed to predict the factors of annual medical expenses and annual medical care use, as well as medical expenses growth. The results indicate that decreasing biological age means lower medical expenses, fewer outpatient and inpatient days, and reduced growth in medical expenses. By quantifying decreases in the factors of medical expenses and medical care use depending on how much BA is reduced, this study can motivate people to become more health-conscious and take care of their physical health. In particular, it is noteworthy that this study is the first attempt to predict medical expenses and medical care use through biological age that reflects health and aging. This study’s analytical findings can remind people that despite the same gender and CA, BA varies considerably from person to person, for which personalized predictions of medical expenses and medical care use are available. Currently, with the aging of the global population, people are growing more and more interested in medical expenses for the elderly population. In these circumstances, numerous research attempts are being made to estimate medical expenses, and the present study, being the first to make BA-based predictions of medical expenses, is expected to push these attempts to new ground.

## References

[1] Yoon SH, Ryu KS, Oh YS, Cho YW, Jin I, Yoo JA, et al. Low birthrates, aging, and the role of finance. Policy, Management Report, 2011;(4):1–255.

[2] Statistic Korea [Internet]. 2021 Senior Citizens Statistics. Available from: https://kostat.go.kr/portal/korea/kor_nw/1/1/index.board?bmode=read&aSeq=403253.

[3] NHIS [Internet]. 2020 National Insurance Statistics. Available from: https://www.hira.or.kr/bbsDummy.do?pgmid=HIRAA020045020000&brdScnBltNo=4&brdBltNo=2313&pageIndex=1#none.

[4] Chung CR. Analysis of the factors that increase medical expenses using the sample cohort data [dissertation]. Seoul: Seoul National University; 2015.

[5] Ministry of Health and Welfare [Internet]. 2019 National Health Accounts. Available from: http://www.mohw.go.kr/react/jb/sjb030301vw.jsp?PAR_MENU_ID=03&MENU_ID=032901&CONT_SEQ=366995.

[6] Lee HS, Yeom YH. A study on medical services by the elderly, medical expenses, and the developmental trajectory and age difference of health outcomes through panel data. Health and Social Welfare Review. 2017;37(2):287–324.

[7] Park HJ, Lee CH, Kim WC, Oh SH. Estimation of medical expenses depending on demographic changes. Journal of Preventive Medicine and Public Health. 1992;25(3):303–311.

[8] Shin JW, Chung HS. Analysis of determinants that dictate household medical outlays. Health Economy and Policy Research. 2007;13(2):97–117.

[9] Chung HS, Song YM. Analysis of the factors that increase covered medical expenses for the elderly and relevant predictions. Health Economy and Policy Research. 2013;19(2):21–38.

[10] Choi BH, Nam SH, Shin YJ. Analysis of the determinants for national medical expenses. Health Policy and Management. 2004;14(2):99–116.

[11] Jacobzone S, Oxley H. Ageing and health care costs. Internationale Politik und Gesellschaft. 2002;(1):137–156.

[12] Atella V, Piano Mortari A, Kopinska J, Belotti F, Lapi F, Cricelli C, et al. Trends in age‐related disease burden and healthcare utilization. Aging Cell. 2019;18(1),e12861. doi: 10.1111/acel.12861 30488641 PMC6351821

[13] Lee KS, Chung HS, Hwang SW, Choi DB, Choi BY, Kim HN, et al. Efficient management of medical expenses for the elderly to cope with an aging society. Korea Institute for Health & Welfare Policy (incorporated association), NHIS. 2017.

[14] Son MS, Kim HK, Lee HS, Choi MK. The longitudinal effect of complex chronic diseases on medical expenses and the prediction of a cut-off point when overburdened medical costs occur. Health Economy and Policy Research. 2018;24(3):49–75.

[15] Kim YR. Relationship between the participation of the elderly in sports and their health status and medical expenses. Sports Science Research. 2006;17(4):125–137.

[16] Lee HH, Park JY. The effect of health screening on personal medical costs. Health Policy and Management. 2014;24(1):35–46.

[17] Buchner F, Wasem J. “Steeping” of health expenditure profiles. The Geneva Papers on Risk and Insurance-Issues and Practice. 2006;31(4):581–599.

[18] Gregersen FA. The impact of ageing on health care expenditures: a study of steepening. The European Journal of Health Economics. 2014;15(9):979–989. doi: 10.1007/s10198-013-0541-9 24271039 PMC4228175

[19] Song SY, Chun HJ, Choi BE. Potential class types of the trajectory of changes in medical expenses for the elderly: their impact on predictors and subjective health. Journal of the Korea Gerontological Society. 2019;39(3):467–484.

[20] Wang C, Li F, Wang L, Zhou W, Zhu B, Zhang X, et al. The impact of population aging on medical expenses: a big data study based on the life table. Bioscience Trends. 2017; 11(6):619–631. doi: 10.5582/bst.2017.01243 29225282

[21] Chao YS, Wu CJ, Chen TS. Risk adjustment and observation time: comparison between cross-sectional and 2-year panel data from the Medical Expenditure Panel Survey (MEPS). Health Information Science and Systems. 2014;2(1):1–9. doi: 10.1186/2047-2501-2-5 25825669 PMC4340859

[22] Choi R, Kang HG. Factors influencing class agreement and medical expenditure by age in South Korea. Medicine. 2018;97:40(e12681). doi: 10.1097/MD.0000000000012681 30290657 PMC6200463

[23] Zhang Y, Lu S, Niu Y, Zhang L. Medical expenditure clustering and determinants of the annual medical expenditures of residents: a population-based retrospective study from rural China. BMJ Open. 2018;8(6):e022721. doi: 10.1136/bmjopen-2018-022721 29934397 PMC6020986

[24] Von Wyl V. Proximity to death and health care expenditure increase revisited: A 15-year panel analysis of elderly persons. Health Economics Review. 2019;9(1):1–16. 30859485 10.1186/s13561-019-0224-z PMC6734245

[25] Lee HY, Kondo N, Oh J. Medical expenditure and unmet need of the pre-elderly and the elderly according to job status in Korea: Are the elderly indeed most vulnerable?. PLoS ONE. 2018;13(3):e0193676. doi: 10.1371/journal.pone.0193676 29570736 PMC5865714

[26] Diehr P, Yanez D, Ash A, Hornbrook M, Lin DY. Methods for analyzing health care utilization and costs. Annual Review of Public Health. 1999;20(1):125–144. doi: 10.1146/annurev.publhealth.20.1.125 10352853

[27] Gregori D, Petrinco M, Bo S, Desideri A, Merletti F, Pagano E. Regression models for analyzing costs and their determinants in health care: an introductory review. International Journal for Quality in Health Care. 2011;23(3):331–341. doi: 10.1093/intqhc/mzr010 21504959

[28] Jia L, Zhang W, Chen X. Common methods of biological age estimation. Clinical Interventions in Aging. 2017;12:759. doi: 10.2147/CIA.S134921 28546743 PMC5436771

[29] Hamczyk MR, Nevado RM, Barettino A, Fuster V, Andres V. Biological versus chronological aging: JACC focus seminar. Journal of the American College of Cardiology. 2020;75(8):919–930. doi: 10.1016/j.jacc.2019.11.062 32130928

[30] Levine ME, Crimmins EM. A comparison of methods for assessing mortality risk. American Journal of Human Biology. 2014;26(6):768–776. doi: 10.1002/ajhb.22595 25088793 PMC4286244

[31] Levine ME. Modeling the rate of senescence: can estimated biological age predict mortality more accurately than chronological age?. Journals of Gerontology Series A: Biomedical Sciences and Medical Sciences. 2013;68(6):667–674. doi: 10.1093/gerona/gls233 PMC3660119 23213031

[32] Yoo J, Kim Y, Cho ER, Jee SH. Biological age as a useful index to predict seventeen-year survival and mortality in Koreans. BMC Geriatrics. 2017;17(1):1–10. 28056846 10.1186/s12877-016-0407-y PMC5217268

[33] Belsky DW, Caspi A, Houts R, Cohen HJ, Corcoran DL, Danese A, et al. Quantification of biological aging in young adults. Proceedings of the National Academy of Sciences. 2015;112(30):E4104–E4110. 10.1073/pnas.1506264112 PMC4522793 26150497

[34] Kang YG, Suh E, Lee JW, Kim DW, Cho KH, Bae CY. Biological age as a health index for mortality and major age-related disease incidence in Koreans: National health Insurance service–health screening 11-year follow-up study. Clinical Interventions in Aging. 2018;13:429. doi: 10.2147/CIA.S157014 29593385 PMC5865564

[35] Soriano-Tárraga C, Giralt-Steinhauer E, Mola-Caminal M, Ois A, Rodríguez-Campello A, Cuadrado-Godia E et al. Biological age is a predictor of mortality in ischemic stroke. Scientific Reports. 2018;8(1):1–8. 29515201 10.1038/s41598-018-22579-0 PMC5841388

[36] Bae CY, Kang YG, Kim S, Cho C, Kang HC, Yu BY, et al. Development of models for predicting biological age (BA) with physical, biochemical, and hormonal parameters. Archives of Gerontology and Geriatrics. 2008;47(2):253–265. doi: 10.1016/j.archger.2007.08.009 17889950

[37] Bae CY, Kang YG, Piao MH, Cho BL, Cho KH, Park YK, et al. Models for estimating the biological age of five organs using clinical biomarkers that are commonly measured in clinical practice settings. Maturitas. 2013;75(3):253–260. doi: 10.1016/j.maturitas.2013.04.008 23642770

